# Phytotoxic Activity of *Myrciaria cuspidata* O. Berg, a Dominant Myrtaceae Woodland Tree Native of Brazil

**DOI:** 10.3390/plants13233293

**Published:** 2024-11-23

**Authors:** Yve V. S. Magedans, Fábio A. Antonelo, Kelly C. S. Rodrigues-Honda, Paula O. S. Ribeiro, Maria E. Alves-Áquila, Arthur G. Fett-Neto

**Affiliations:** 1 Plant Physiology Laboratory, Center for Biotechnology, Federal University of Rio Grande do Sul (UFRGS), Porto Alegre 91501-970, Brazil; yve.silva@gmail.com (Y.V.S.M.); fabioantonioantonelo@gmail.com (F.A.A.); paulaodiles42@gmail.com (P.O.S.R.); 2 Plant Physiology Laboratory, Department of Botany, Federal University of Rio Grande do Sul (UFRGS), Porto Alegre 91501-970, Brazil; honda9@hawaii.edu (K.C.S.R.-H.);

**Keywords:** *Myrciaria*, allelopathy, germination, growth, phenolics, woodlands

## Abstract

Limited phytodiversity and regeneration rates occur in some of the southern Brazilian formations known as the Myrtacean Woodlands. Data on phytotoxicity, chemical composition, and allelopathic potential of *Myrciaria cuspidata* O. Berg, a dominant species in such woodlands, is missing. In this study, both the chemical composition and phytotoxic activity of an aqueous extract (AE) from *M. cuspidata* leaves were investigated. Target plants were the model species *Lactuca sativa* L. and the weed *Bidens pilosa* L. Germination rates, seedling growth, and phenotypic responses of target species were assessed following AE application to determine the inhibitory capacity of *M. cuspidata* leaf extract. Germination of *L. sativa* was reduced and delayed in the presence of AE. Strong inhibition of germination was recorded in *B. pilosa* achenes under the same treatment. Pre-germinated seedlings of *L. sativa* were essentially not affected by AE, whereas those of the weed showed some negative developmental responses. Overall, inhibitory responses were consistent both in vitro and in soil substrate. Detrimental effects were most apparent in roots and included tip darkening and growth anomalies often preceded by loss of mitochondrial viability. AE proved rich in phytotoxic phenolic compounds including quercetin, gallic and tannic acid. To sum up, AE shows potential as an environmentally friendly pre-emergence bioherbicide of low residual effect and minor environmental impact. Experimental data in laboratory conditions were consistent with potential allelopathic activity of this tree, as inferred from field observations of dominance in the Myrtaceae Woodlands.

## 1. Introduction

The search for alternative less-toxic sources of herbicides has been an active area of investigation in the last decades. The application of bioactive natural compounds might be a useful tool to prevent environmental damage and artificial selection of resistant weeds in different crops [1].

Allelochemicals are natural compounds produced by several species of plants and released in the environment that are capable of chemically interfering with the development of adjacent plants and/or microorganisms. This interference takes place during the establishment of an ecological interaction that was coined allelopathy by Hans Molisch in the mid-thirties [2]. Of the main broad classes of specialized metabolites (terpenes, phenolics, and nitrogen-containing compounds), shikimate- and some isoprenoid-derivative molecules have frequently been identified as the major allelochemical entities, in part as a function of their relative solubility in water. Allelochemical production can be influenced by an array of biotic and abiotic factors, such as water availability, nutritional imbalance, light quality (e.g., UV radiation, solar irradiance, red:far red ratio), extreme temperatures, salinity, pathogens, herbivores, and symbiotic microorganisms. Not surprisingly, the concentration and activity of specialized metabolites produced by plants often vary seasonally [3].

A great deal of information is available on allelopathy interactions in temperate zones [4]. However, the proposed models for temperate forests may not be necessarily applicable to tropical and subtropical environments. Therefore, studies of this nature in diverse types of vegetation are needed.

Species of Myrtaceae show several biological activities such as antioxidant, anti-inflammatory, hypoglycemic, anticholinesterase, antiplasmodium, antibacterial and antifungal properties. Such activities are often due to the presence of several bioactive specialized metabolites, notably among them phenolics and terpenes, particularly the former [5]. Several genera of Myrtaceae (*Eucalyptus*, *Callistemon*, *Eugenia*, *Blepharocalyx*, *Campomanesia*, *Psidium*, *Myrcia*) have been investigated regarding their allelochemical effects [6,7,8,9,10,11,12,13].

**Figure 1:**
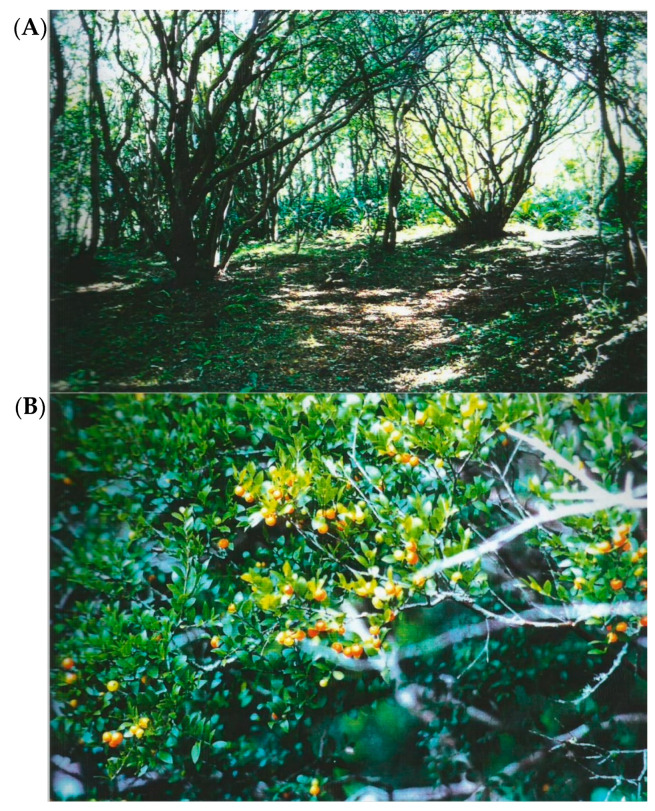
General view of a portion of Myrtacean Woodland, showing the scarce understory vegetation (**A**). Aspect of fruiting branch of *M. cuspidata* (**B**). Photo credit: Kelly Cristine Rodrigues-Honda.

In southern Brazil, early field observations indicated that understory regeneration is less effective in the Myrtacean Woodlands [14] than in other syntopic natural formations in rocky outcrops of granitic hills commonly found in the region of Porto Alegre (Rio Grande do Sul State, approximately 30°03′ S e 51°07′ W). The overall aspect of these formations is consistent with such features (Figure 1A). The Myrtacean Woodlands are mostly located in the lower portions of these hills. Furthermore, soil samples removed from sites where this vegetation is established and mixed with substrate for growing some crop species inhibited their germination and growth [15].

*Myrciaria cuspidata* O. Berg is a native species from Brazil known as “camboim”, one of the dominant species in the Myrtacean Woodlands (Figure 1). It belongs to Myrteae, the largest tribe of the subfamily Myrtoideae [16]. Features of *M. cuspidata* include arborescent habit, opposite apiculate leaves, bisexual flowers, and one-seeded berry fruits [17,18]. The analysis of *M. cuspidata* leaves indicated that chemical defenses are likely the primary mechanism protecting the foliage from herbivory [19]. To the best of our knowledge, however, there is no report available to date on *M. cuspidata* leaves chemical composition or allelopathic effects.

Low-diversity plant communities such as the Myrtacean Woodlands are characterized by limited success in the establishment and fixation of various plant species. Chemical entities with potential as bioherbicides may be at play in shaping these types of vegetation. The hypothesis of this study was that leaf aqueous extracts of *M. cuspidata* have allelopathic activity, inhibiting germination and growth of certain plant species. Considering the relevance of new sources of environmentally friendly herbicides and the need to better understand the dynamics of low-diversity plant communities, this work evaluated the allelopathic potential of leaves of *M. cuspidata.* This study aimed to evaluate the phytotoxic activity of *M. cuspidata* leaf aqueous extract on *Lactuca sativa* L. (lettuce) and on the weed *Bidens pilosa* L. through germination and growth bioassays.

## 2. Results

### 2.1. Chemical Characterization of AE

AE pH value varied between 5.0 and 7.0 in all cases. Osmotic potential values did not surpass −0.11 MPa (Supplementary Table S1). The initial extract yield was 0.015 g/mL.

**Figure 2:**
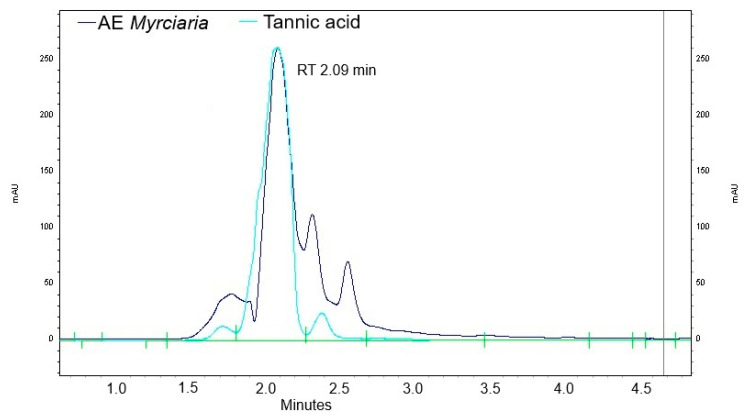
Chromatogram of *Myrcyaria cuspidata* leaf aqueous extract at 0.04 g/mL (dark blue) and authentic tannic acid at 100 µg/mL (light blue). Detection set at 280 nm.

Thin layer chromatography showed the presence of tannic acid (Supplementary Figure S1), which was further confirmed by high-performance liquid chromatography (HPLC) (Figure 2). Tannic acid concentration in the aqueous extract was 0.01 ± 0.003% (*w*/*v*) (n = 3), as assessed by HPLC. In contrast, rutin was not detected. Colorimetric determinations showed AE contained 4.92 ± 0.22 mg/mL of total phenolic compounds. Total tannins and total flavonoids in the extract were found at 0.58 ± 0.19 mg/mL and 0.26 ± 0.12 mg/mL, respectively (Supplementary Table S1). LCMS data confirmed the presence of flavonols (quercetin, quercitrin) and phenolic acids (gallic acid, ellagic acid) in AE (Supplementary Table S2).

### 2.2. Qualitative Overview of Phytotoxicity Tests

Overall, AE inhibited the germination of both target species more effectively than the growth of pre-germinated seedlings. This response was similar in Petri dishes and soil substrate. Although significant in both cases, in Petri dishes, the extent of germination inhibition by AE was much higher for *B. pilosa* than for *L. sativa*. In soil, the inhibition of germination by AE was similar for both species. Germinated seedlings of *L. sativa* derived from achenes exposed to AE had damaged root tips that became dark and lost viability soon after germination. Seedlings of both target species derived from AE-exposed achenes had reduced growth. Whereas non-germinated *L. sativa* achenes exposed to AE lost viability, the same did not apply to *B. pilosa* ones. Pre-germinated seedlings treated with AE had reduced length in Petri dishes for both species, whereas in soil, only *B. pilosa* seedlings showed significantly lower dry biomass. This contrasted with increased dry biomass of the same seedlings observed under the AE exposure in Petri dishes. In all cases, the negative effect of AE was most apparent in roots. In the sections below, each one of these data sets is separately and quantitatively presented in detail.

### 2.3. Effects of AE on In Vitro Germination

**Figure 3:**
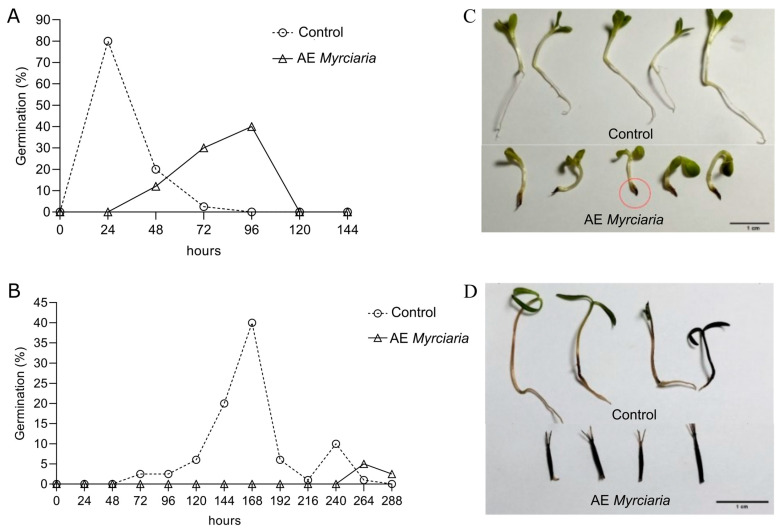
Germination of *L. sativa* (**A**,**C**) and *B. pilosa* (**B**,**D**) achenes in Petri dishes containing water (control) or *M. cuspidata* leaf aqueous extract at 4% (*w*/*v*). Germination time course was recorded at 24 h intervals. Darkening of germinated *L. sativa* seedlings roots whose achenes were in the presence of leaf extract is visible (red circle).

The aqueous extract of *M. cuspidata* negatively affected the germination of both *L. sativa* and *B. pilosa* in Petri dishes. Significantly slower germination and reduced germinability were observed. The germination peak of *L. sativa* achenes was delayed from 24 to 96 h, and the final germination rate decreased from 100% to 74%. The same parameters changed from 168 to 264 h and 74 to 6% in the experiments with *B. pilosa* (Figure 3A,B; Supplementary Figure S2). Representative germinated seedlings of *L. sativa* and *B. pilosa* are shown in Figure 3C,D, respectively. Although most *L. sativa* achenes germinated in the presence of AE, shorter roots and dark root tips developed in the resulting seedlings (Figure 3C).

Ungerminated *L. sativa*-treated achenes exposed to AE were not viable at the end of the experiment, showing no staining in the TTC (2,3,5-triphenyltetrazolium chloride) test. Embryos from achenes of *B. pilosa* that did not germinate for 12 days were excised and subjected to the TTC test. In addition, a set of abundantly washed achenes was allowed to germinate in Petri dishes with water. Both the viability and germinability tests indicated that unlike *L. sativa*, *B. pilosa* achenes remained viable after the application of AE (Supplementary Figure S3), despite its strong inhibitory effect on germination (Figure 3B; Supplementary Figure S2).

### 2.4. Effects of AE on In Vitro Seedling Growth

**Figure 4:**
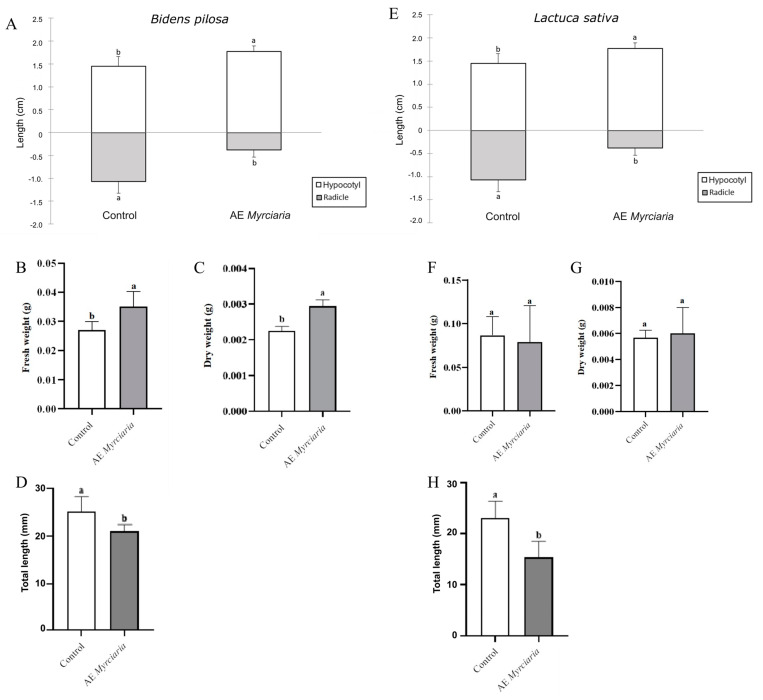
Growth of *B. pilosa* and *L. sativa* in Petri dishes. Pre-germinated seedlings were grown in *M. cuspidata* leaf aqueous extract at 4% (*w*/*v*) or water (control). Data were obtained at the end of the experiments (6 days for *L. sativa* and 12 days for *B. pilosa*). Radicle and hypocotyl elongation (**A**,**E**), comparisons valid within each organ), fresh weight (**B**,**F**), dry weight (**C**,**G**), total seedling length (**D**,**H**). Control and treated groups were compared using a *t*-test (*p* ≤ 0.05). Bars not sharing a letter are significantly different.

Growth parameters were evaluated in pre-germinated seedlings transferred to Petri dishes and treated with AE. Unexpectedly, both fresh and dry weights of pre-germinated *B. pilosa* plants increased significantly compared to control after 6 days of treatment (Figure 4B,C). However, a decrease in total length was observed due to root elongation inhibition (Figure 4A,E). Interestingly, this treatment promoted root-negative gravitropism in approximately 38% of seedlings (Supplementary Figure S4).

*L. sativa* pre-germinated seedlings exposed to AE did not exhibit differences in weight compared to control ones (Figure 4F,G). Despite the presence of longer hypocotyls under AE exposure, a decrease in total seedling length was recorded due to radicle length reduction (Figure 4H), as observed in *B. pilosa* roots. Apparent darkening and death of root tissues (particularly root tips) after exposure to AE were visible (Supplementary Figure S5), akin to what was observed for seedlings germinated in the same conditions. Viability tests using TTC indicated that roots of *L. sativa* seedlings exposed to AE were dead by the end of the experiment (Supplementary Figure S5). Furthermore, a test using Janus Green staining revealed mitochondrial damage in the treated roots, as evidenced by the absence of pink-colored mitochondrial aggregates after 24 and 48 h of treatment (Supplementary Figure S6).

### 2.5. Effects of AE on Germination and Seedling Growth in Soil

**Figure 5:**
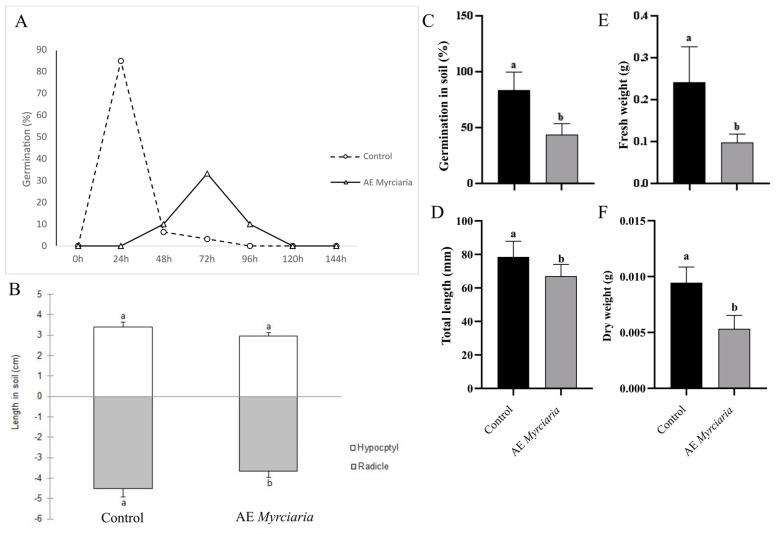
Germination and growth of *L. sativa* on the solid substrate. *M. cuspidata* leaf aqueous extract 4% (*w*/*v*) or water (control) sprayed twice—at day 0 and 48 h. (**A**)—Germination time course recorded at 24 h intervals; (**B**)—Radicle and hypocotyl elongation (comparisons valid within each organ); (**C**)—Germination percentage in soil; (**D**)—total length of plants; (**E**)—fresh weight; (**F**)—dry weight. Control and treated groups were compared using *t*-test (*p* ≤ 0.05). Bars not sharing a letter are significantly different.

**Figure 6:**
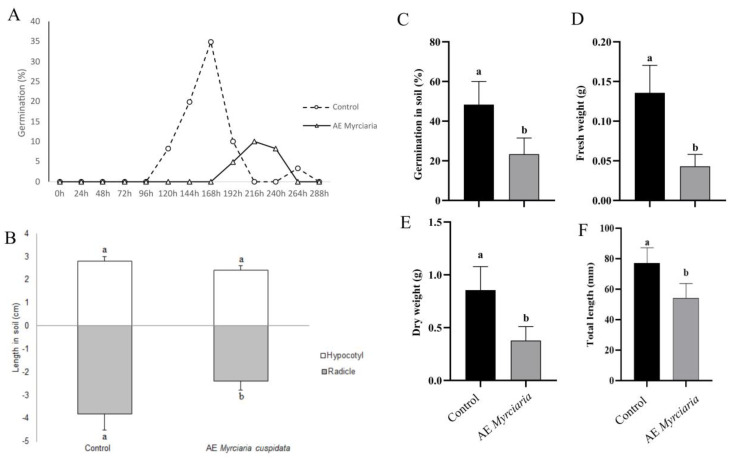
Germination and growth of *B. pilosa* on solid substrate. *M. cuspidata* leaf aqueous extract 4% (*w*/*v*) or water (control) were sprayed in plants twice—at day 0 and 48 h. (**A**)—Germination time course recorded at 24 h intervals; (**B**)—Radicle and hypocotyl elongation (comparisons valid within each organ); (**C**)—Germination percentage in soil; (**D**)—Fresh weight and (**E**)—Dry weight; (**F**)—Total length of plants. Control and treated groups were compared using a *t*-test (*p* ≤ 0.05). Bars not sharing a letter are significantly different.

When germination was evaluated directly on a solid substrate, AE was sprayed on the achenes at the establishment of the experiment and after 48 h. A reduction in the percentage of germination was seen for *L. sativa-* (Figure 5A,C) and *B. pilosa-*treated (Figure 6A,C) achenes. Treated achenes of *L. sativa*, however, germinated earlier in soil compared to Petri dishes (72 versus 96 h, respectively). Overall, growth parameters were negatively affected by spraying AE for both species (Figure 5 and Figure 6). In contrast, extract application on pre-germinated seedlings transferred to a solid substrate did not significantly affect the growth parameters (Supplementary Figures S7 and S8), except for a reduction in dry weight of AE-exposed *B. pilosa* seedlings (Supplementary Figure S8B).

## 3. Discussion

The pH values of AE (5.0 to 7.0) were out of the inhibitory range for germination and growth of both tested species [20,21]. Likewise, the water potential of AE was—0.11, outside the phytotoxic range for both *L. sativa* [22,23] and *B. pilosa* [21]. These data support specific effects of AE on target plants rather than mere general negative impacts of physical variables.

The activity of plant extracts often relies on the solvent used to prepare the extracts. However, since water is the most common solvent in field conditions, a possible allelopathic effect of *M. cuspidata* leaves on understory species is likely. Moreover, the potential—of AE in weed management and control practices may also be facilitated by using this non-toxic, relatively cheap, and abundant solvent.

Overall, AE inhibited the germination of both target species more effectively than the growth of pre-germinated seedlings. This trend was similar in Petri dishes and soil substrate. Some differences observed between the tested species exposed to AE (e.g., higher degree of germination inhibition in *B. pilosa* but without the loss of viability compared to *L. sativa*) may reflect distinctive features related to extract penetration, cell sensitivity, and critical length of exposure. This observation is in line with the complexity of allelochemical interactions, which often show some taxa-dependent responses.

Allelochemical effects on germination and development can be related to several events, such as alterations in cell membrane permeability and redox balance, along with modifications in gene expression and protein profiles. These alterations often result in impaired cell growth [24]. Strong inhibition on radicle growth was recorded, as well as expression pattern changes in genes related to root development (*SHR* and *PHB*) in sorghum seeds treated with hydroalcoholic extracts of *Myrcia guianensis* (Aubl.) DC. leaves [10]. Although similar gene expression modifications may be caused by AE in radicles of target species, root darkening was not reported [10], suggesting differences in mechanisms or intensity of root growth inhibition induced by *M. cuspidata*.

Morphological and ultrastructural alterations in plant roots, including stiffening and/or darkening of tips, are common signs of allelochemical effects. These changes are related to tissue disorganization and reduced development at the affected site, often indicating damage to organelles (e.g., mitochondria and endoplasmic reticulum), plasma membrane integrity, and cell wall structure [25]. In the present study, exposure to AE resulted in root cell mitochondria function impairment, as supported by the TTC and Janus Green data on *L. sativa*. Indeed, the loss of mitochondrial function was evident 24 h after AE treatment, preceding root darkening by about the same amount of time.

Members of Myrtaceae are often characterized as phytotoxic, a feature at least partially related to the widespread presence of phenolics in their composition [26,27,28,29]. Chemical analyses showed the presence of phenolics in AE, as assessed by quantification of total phenolics, flavonoids, and tannins. The concentrations recorded of these components were comparable to those reported for similarly obtained leaf aqueous extracts from other woody species such as *Pistacea lentiscus* [30]. Evidently, even though the focus of the present study was on phenolics because of their major presence in *M. cuspidata*-related taxa, the participation of other AE metabolites in its phytotoxic activity cannot be ruled out.

Both TLC and HPLC analyses confirmed the presence of tannic acid, which is commonly accumulated in *Myrciaria* [29]. Although flavonoids were present in AE, rutin was not detected, possibly due to its limited solubility in water [31]. In contrast, tannins are highly water-soluble [32] and more readily extracted with this solvent from the leaf powder.

Phenolics present in AE are probably involved in the observed phytotoxic effect on *L. sativa* and *B. pilosa* achenes and plantlets. Gallic acid and quercetin have been shown to be allelochemicals [33]. Tannic acid was identified as a wheat root growth inhibitor present in extracts of the vine *Ampelocissus latifolia* Roxb. (Planch) [34]. The tannic acid concentration found in AE was twice that found to significantly inhibit root growth of wheat seedlings by the last authors. In addition, the concentration of tannic acid in AE was approximately an order of magnitude higher than that required to cause mitochondrial viability loss in animal cell cultures [35]. Clearly, the chemical profile of AE is consistent with allelopathic properties.

Tannins are well known for binding and precipitating proteins, causing the interruption of important physiological processes. Among other properties, tannins can inhibit the activity of several enzymes, such as amylases, proteases, lipases, pectinases, and cellulases [32]. All these enzymes are relevant for reserve mobilization and growth during germination and seedling establishment. The affinity of enzymes for tannins varies. These phenolics are actual enzyme modifiers rather than only inhibitors, as they can even increase some enzyme activities. Moreover, tannins can interact with other organic non-protein N metabolites such as arginine, nitrogen bases, and polyamines [36].

Loss of mitochondria viability in root tips of *L. sativa* seedlings exposed to AE may also have been influenced by its phenolics. Tannic-acid-induced damage to mitochondrial functions and integrity in animal cell cultures involves decrease in mitochondrial membrane potential and ATP production [35]. Similar cytotoxic activity in mammalian cancer cells via mitochondrial damage has been reported for gallic acid [37]. At least in part, phytotoxicity mechanisms of AE may also include mitochondrial impairment consistent with root tip darkening and phenolic composition. However, caution must be exercised in extrapolating mechanisms described for animal cells to plants.

The occurrence of negative gravitropism in a significant portion of *B. pilosa* seedlings transferred to plates with AE is likely to impact further growth. Indeed, soil tests showed that the dry weight of seedlings was significantly reduced when these were treated with AE. Although gravitropic response has multiple components and specific genes playing roles in organ directional growth, a key element is the establishment of auxin gradients between root sides. These gradients are mainly created by efflux transporter location, organization, and function [38]. It is possible that AE may have interfered with the redistribution and activity of auxin transporters, thereby disrupting the normal positive gravitropic response in roots. In fact, roots proved to be the main target of AE phytotoxicity in all the different experiments carried out. The presence of flavonoids in AE could be at least partly responsible for such effects since some of these phenolics (including quercetin) are known as inhibitors of auxin transporters and modulators of auxin homeostasis [39].

Similarly to growth regulators, allelochemicals can have different effects on plants depending on their concentrations. In fact, at low concentrations, allelochemicals can be beneficial to the expression of some morphological parameters [40]. Those effects can vary depending on the developmental stage, evaluated structure, or organ [20,41]. Indeed 4% aqueous *M. cuspidata* extract promoted hypocotyl elongation, inhibited radicle extension and overall seedling length, but promoted biomass accumulation of pre-germinated *B. pilosa* in Petri dishes. There is no obvious mechanistic explanation for growth stimulation in *B. pilosa* exposed to AE. However, it may be possible that auxin homeostasis modulation by flavonols of the extract [39] could promote biomass increase under specific experimental conditions. Flavonols can not only affect auxin efflux transporters but also scavenge reactive oxygen species (ROS) due to their antioxidant properties. This last feature may modulate not only growth but also other processes dependent on ROS balance and signaling, such as stomatal aperture [42].

The results observed in the bioassays conducted in Petri dishes and soil substrate under laboratory conditions are in line with field observations. In the understory and surroundings of *M. cuspidata*, the presence of other plants is sharply reduced (Figure 1), thereby suggesting its allelopathic activity. Field studies could provide further understanding of the environmental dynamics of this interaction, aiming at contributing to the elucidation of community shaping and potential ecological management of *M. cuspidata* and related species from the Myrtacean Woodlands. On the other hand, the use of AE as a potential pre-emergence bioherbicide seems promising, considering its significant germination and early growth phytotoxicity both in in vitro and in soil substrate. The low residual effect due to water solubility and biodegradability may also be advantageous for agrobiological applications. Follow-up studies should address several remaining gaps, such as bioactivity-guided fractionation of AE components, expansion of target plants (notably weed and crop species panels), field-based experiments, extract shelf life and biodegradation profile, molecular mechanisms of action, and delivery systems (e.g., spraying, encapsulation, use in herbicidal mixtures).

## 4. Materials and Methods

### 4.1. Plant Material and Preparation of Extracts

*Myrciaria cuspidata* O. Berg (Myrtaceae) leaves were collected at Morro Santana, Porto Alegre, RS, Brazil (30°03′ S 51°07′ W) during the summer of 2023/2024. A voucher specimen (122551) was deposited at the ICN Herbarium (UFRGS, Porto Alegre, Brazil). Leaves were dried for a week at room temperature (25 ± 3 °C) under shade. Experiments were carried out with pooled leaf batches obtained from at least three different individuals. Propagules of *Lactuca sativa* L. cv. White Boston (Asteraceae) were purchased in local markets for all experiments. Achenes of *Bidens pilosa* (L.) (Asteraceae) were collected at Dois Vizinhos, Paraná, Brazil (25°43′ S 52°59′ W) (voucher specimen deposited in ICN Herbarium—212066). 

Dried leaves were powdered and extracted with boiling distilled water (0.04 g/mL). The macerations were kept at room temperature for 24 h. Extracts were then filtered through cheesecloth and centrifuged at 1300× *g* for 10 min. Water was completely removed by lyophilization. Next, extract residues were resuspended in distilled water at 4% (*w*/*v*) prior to use. This preparation constituted the *M. cuspidata* leaf aqueous extract, hereafter abbreviated as AE. Extract concentration was based on preliminary experiments and previous experience with other tree species [43].

### 4.2. Chemical Characterization

Omotic potential of the AE was estimated by Chardakov’s method [44], and pH was measured with a potentiometer. The extract yield was also evaluated by weighing, following complete lyophilization. Three extracts of independent biological replicates were analyzed.

Total phenolic concentration was measured as previously described [45], with minor modifications. Briefly, AE was acidified with 0.1 N HCl (920 µL). Subsequently, 20% Na_2_CO_3_ (40 µL) and Folin–Ciocalteu reagent (10 µL) were added. The samples were incubated in a water bath at 100 °C for 1 min and immediately cooled on ice. Spectrophotometric readings were taken at 750 nm. Pyrogallol was used as the standard. Total tannin amount was obtained as the concentration difference between samples of untreated AE and samples of the same that were submitted to tannin precipitation with 2% gelatin [46]. Three extracts of independent biological replicates were analyzed.

Total flavonoid concentration was also estimated [47]. Briefly, the extract was diluted and mixed with an equal volume of 2% aluminum chloride hexahydrate. Subsequently, 5% sodium acetate was added. After 15 min, absorbances were read at 440 nm. Total flavonoids were expressed as mg of quercetin equivalents per mL. Three extracts of independent biological replicates were analyzed.

The phenolic profile of AE was initially evaluated by thin-layer chromatography (TLC). The mobile phase was ethyl acetate:formic acid:acetic acid:water (20:2.2:2.2:5.2). Silica gel aluminum plates (F254, Supelco, Darmstadt, Germany) measuring 7 cm in length were used as stationary phase. Plates were revealed under UV light (254 nm). Authentic rutin (rutin hydrate, minimum 95% HPLC grade—Sigma, St. Louis, MI, USA) and tannic acid (Merck, Darmstadt, Germany) were used as standards [48]. Three extracts of independent biological replicates were analyzed.

Phenolics in AE were also examined using high-performance liquid chromatography (HPLC). The equipment used for analysis was a Surveyor System (Thermo Scientific, Waltham, MA, USA) with a UV–vis Diode Array Detector. The isocratic chromatographic method had a mobile phase composed of methanol 70% + 0.1% trifluoroacetic acid and a flow rate of 0.8 mL/min; the stationary phase was a Waters Spherisorb C8 reverse phase analytical column (150 × 4.6 mm) (Milford, MA, USA), and main detection was set at 280 nm. Three extracts of independent biological replicates were analyzed. The sample injection volume was 20 μL. The extract concentration was 0.04 g/mL. External standard curves with authentic tannic acid and rutin were prepared for the detection and quantification of these compounds in AE. Identification was based on retention time, UV spectra, and co-chromatography [49].

Further characterization of AE was performed by UHPLC-QTOF-MS-(UPLC model Nexera 2 Shimadzu, Tokyo, Japan and QTOF-MS model Bruker Daltonics, Billerica, MA, USA) as previously described [50]. Briefly, samples were diluted to 5 mg/mL, and 1 μL was injected onto a Shimadzu (Tokyo, Japan) Shim-Pack XR-ODS-III column (C18, 2.2 μm, 2.0 × 150 mm) at 40 °C. The flow rate was set at 400 μL/min. The mobile phases were 0.1% formic acid in MilliQ water (A) and acetonitrile (B). Runs started in isocratic mode of 5% A for 5 min, followed by a linear gradient to 100% B in 40 min and held at 100% B for 5 min. Ion-source parameters for mass analyses were set to 500 V end plate offset, 4500 V capillary voltage, 3.0 bar nebulizer pressure, 8 L/min dry gas flow, and 200 °C of temperature. Mass spectra were acquired at m/z 100–1500. Data-dependent fragment spectra were recorded with a collision energy range between 15 and 60 eV. Mass calibration was achieved by initial ion-source infusion of 20 μL 1 mM sodium formate in 50% 2-propanol and post-acquisition recalibration of the raw data. Chromatographic dissection and subsequent peak deconvolution and formula determination according to exact mass and isotope pattern (MS1) were used for compound detection, and identification was by comparison to public databases [50].

### 4.3. Germination Analysis and Evaluation of Seedlings Growth in Petri Dishes

Ten achenes of *L. sativa* per Petri dish were directly imbibed in water (control) or AE, totaling 100 achenes per treatment. Ten achenes per Petri dish (a total of 50 achenes per treatment) were used to test the germination of *B. pilosa*. The germination criterion was radicle emergence. Experiments were conducted in a growth chamber with a temperature of 20 ± 2 °C and 8 h of dim light per day (PAR—photosynthetically active radiation of 20 μmol·m^−2^·s^−1^). *L. sativa* experiments were monitored for 144 h and those of *B. pilosa* for 288 h. Germination curves were plotted, and final germinability was recorded. 

For growth evaluation, achenes from *L. sativa* and *B. pilosa* were pre-germinated in distilled water. Immediately after radicle protrusion, 10 seedlings per dish (n = 100 per treatment) displaying circa 1 mm as standard root length were placed on Petri dishes containing distilled water (control) or AE. The same procedure was applied to *B. pilosa* seedlings. At the end of 6 days, hypocotyl and radicle lengths were measured. Seedlings fresh and dry biomass (n = 50) were also recorded at the end of the experiment. Dry mass was obtained by incubation at 60 °C until constant mass. 

### 4.4. Germination and Growth Analysis on Soil-like Substrate Sprayed with Leaf Extract

*L. sativa* or *B. pilosa* achenes (ten achenes per pot, totaling 60 for each species) were pre-germinated in Petri dishes and then transferred to 200 mL plastic pots containing solid substrate (garden soil:sand—1:1 w/w). Six days after germination, the plants were transferred to the vases and maintained for 7 days. Experiments were carried out in a growth room with 16 h light photoperiod and PAR of 140 μmol·m^−2^·s^−1^, temperature between 20 and 25 °C, and air humidity ranging from 60 to 75. Pots were bottom watered with distilled water every two days.

Seven days after the transference to solid substrate, AE was sprayed on the potted plants, roughly to the point of imminent run off (approximately 0.05 mL per cm^2^). The extract was reapplied 48 h after the first application. Control samples were sprayed with distilled water. The percentage of survival in soil, total length, fresh and dry weight were recorded after 15 days of the first application.

To evaluate the effect on germination, *B. pilosa* or *L. sativa* achenes were directly germinated in 200 mL plastic pots containing a mixture of garden soil and sand (1:1 w/w). Ten achenes per pot (n = 60) were seeded, and soon after, each vase was sprayed with AE (circa 0.05 mL/cm^2^). The extract was reapplied 48 h after the first application. Control samples were sprayed with distilled water. Plants were bottom watered every two days with distilled water. Percentage of germination, total length of plantlets, fresh and dry weight were obtained 288 h or 144 h after the experiment began for *B. pilosa* and *L. sativa*, respectively. Germination curves were plotted, and final germinability was recorded.

### 4.5. Viability Analysis

To test cell viability after experiments, a 0.1% solution of 2,3,5-triphenyltetrazolium chloride (TTC) was used. Pre-moistened achenes or germinating seedlings were soaked in TTC in the dark, and incubated at 30 °C for at least 1 h [51]. Next, plant material was observed under a light microscope.

Mitochondrial viability in *L. sativa* roots was evaluated with Janus Green staining. Reduction of the dye by active mitochondrial dehydrogenases yields a pink color. Achenes were pre-germinated in water. Seedlings with a 2 mm radicle were transferred to new plates containing either AE or water (control). After 24 and 48 h of incubation in treatments, the roots were stained with Janus Green for 12 h and observed under a light microscope [52].

### 4.6. Statistical Analysis

For germination assays, the following statistical tests were used: randomness, normality, and Bartlett (variance homogeneity). Analysis of Variance (ANOVA) followed by Tukey test was used for radicle growth (data transformed to 1/square root); Kruskal–Wallis followed by Mann–Whitney, for hypocotyl elongation. The significance level for all analyses was set at 5%. As for solid substrate experiments, radicle growth data were transformed to log 10 before statistical analysis. Experiments comparing two treatments were analyzed with a *t*-test (*p* < 0.05). A Shapiro–Wilk normality test was applied to confirm the normality of the data distribution. Statistical analysis was performed using GraphPad Prism 8.0. Experiments had from 3 (biochemical data) to 10 (germination and growth data; see above for each case and units per replicate) biological replicates. Experiments were also independently repeated twice.

## 5. Conclusions

*M. cuspidata* aqueous extract has potential as a pre-emergence/early growth bioherbicide. The allelopathic profile hinted from field observations can be helpful for identifying bioherbicidal potential. The phytotoxic activity appears related to phenolics, possibly tannins and flavonoids. Roots, particularly meristematic tips, are the main target organs of this extract, and mitochondrial viability loss likely plays a role in the phytotoxicity mechanism. Future studies are needed to investigate the effects of the extract on a wider panel of plant species, both crops and weeds. Fractionation-guided bioactivity will be instrumental in pinpointing key active components for subsequent bioherbicide product development.

## Data Availability

The original contributions presented in this study are included in the article/Supplementary Material, and further inquiries can be directed to the corresponding author.

## Supplementary Materials

The following supporting information can be downloaded at: https://www.mdpi.com/article/10.3390/plants13233293/s1, Supplementary Table S1. Characteristics of *Myrciaria cuspidata* O. Berg aqueous extract. Supplementary Table S2. Phenolics identified by LCMS in *Myrciaria cuspidata* O. Berg aqueous extract. Supplementary Figure S1. Thin layer chromatography of leaf aqueous extracts. 1—Tannic acid (220 µg). 2—Rutin (220 µg). 3 and 4—*M. cuspidata* aqueous leaf extract at 4% (*w*/*v*) (70 µg). Supplementary Figure S2. Final germination percentage *L. sativa* and *B. pilosa* on Petri dishes containing *M. cuspidata* leaf aqueous extract 4% (*w*/*v*) or water (control). Treatments were compared using *t*-test (*p* ≤ 0.05). Supplementary Figure S3. Excised embryos from achenes of *B. pilosa* after 12 days in Petri dishes with water (control just germinated, right) or *M. cuspidata* leaf aqueous extract at 4% (*w*/*v*) (AE ungerminated, left, same time after sowing of control achenes) subjected to the triphenyltetrazolium chloride test. Alternatively, achenes ungerminated in AE were allowed to germinate after abundant washing in distilled water (right). Supplementary Figure S4. *B. pilosa* seedlings pre-germinated in Petri dishes exposed to *M. cuspidata* leaf aqueous extract at 4% (*w*/*v*). After radicle protrusion, root of seedlings transferred to plates with 4% (*w*/*v*) *Myrcyaria cuspidata* aqueous leaf extract often showed negative gravitropism (red circles). Supplementary Figure S5. Root damage in *L. sativa* seedlings. Achenes were pre-germinated and transferred to Petri dishes under control conditions (A) and treated with *M. cuspidata* leaf aqueous extract at 4% (*w*/*v*). (B). A tetrazolium viability test on the roots was performed 6 days after treatment. Supplementary Figure S6. Mitochondrial damage in *L. sativa* roots. Achenes were pre-germinated and transferred to Petri dishes with water (control) (A,B) or 4% (*w*/*v*) leaf aqueous extract of *M. cuspidata* (C,D). Root mitochondrial viability test with Janus Green was performed at day 1 and 2 after transfer to water (A,B) or extract treatment (C,D), respectively. Active mitochondria appear pink, whereas non-functional organelles stain green. Supplementary Figure S7. Growth of pre-germinated *L. sativa* seedlings on solid substrate. Seedlings were sprayed twice (0 and 48 h) with water (control) or *M. cuspidata* leaf aqueous extract at 4% (*w*/*v*). Plants were harvested after 15 days from the first application of the treatments. A—Survival of plants in soil; B—Total length; C—Fresh weight; D—Dry weight; E—Length of hypocotyl and radicle. Control and treated groups were compared using *t*-test (*p* ≤ 0.05). Supplementary Figure S8. Growth of pre-germinated *B. pilosa* seedlings on solid substrate. Seedlings were sprayed twice (0 and 48 h) with water (control) or *M. cuspidata* leaf aqueous extract at 4% (*w*/*v*). Plants were harvested after 15 days from the first application of the treatments. A—Survival of plants in soil; B—Total length; C—Fresh weight; D—Dry weight; E—Length of hypocotyl and radicle. Control and treated groups were compared using *t*-test (*p* ≤ 0.05).
