# Supplementary material for: Phytotoxic Activity of Myrciaria cuspidata O. Berg, a Dominant Myrtaceae Woodland Tree Native of Brazil
Source: Plants (Basel). 2024 Nov 23;13(23):3293. doi: 10.3390/plants13233293 (PMC11644669; doi:10.3390/plants13233293)
Supplement: Supplementary file 1 [file plants-13-03293-s001.zip › plants-3253370-supplementary.pdf]

Supplementary table S1. Characteristics of *Myrciaria cuspidata* O. Berg aqueous extract.

**Parameter**	**Value**
Plant : solvent ratio (% w/v)	4
Yield (g/mL)	0.015
Concentration (% w/v)	4
pH	5 to 7
Osmotic potential (MPa)	- 0.11
Total phenolics (mg/mL pyrogallol equivalents)	4.92 ± 0.22
Total flavonoids (mg/mL quercetin equivalents)	0.26 ± 0.12
Total tannins (difference post-gelatin precipitation of total phenolics)	0.58 ± 0.19

Supplementary table S2. Phenolics identified by LCMS in *Myrciaria cuspidata* O. Berg aqueous extract.

**Adduct type**	**Retention time (min)**	**Measured m/z**	**Ion formula**	**m/z**	**Error (ppm)**	**Compound name**
[M-H]-	7.3	447.0925	$C_{21}H_{19}O_{11}$	447.0933	1.8	quercitrin
[M-H]+	7.3	303.0507	$C_{15}H_{11}O_{7}$	303.0499	-2.5	quercitin
[M-H]-	7.2	169.0144	$C_{7}H_{5}O_{5}$	169.0142	-0.8	gallic acid
[M-H]-	0.9	300.998	$C_{14}H_{5}O_{8}$	300.9990	3.2	ellagic acid

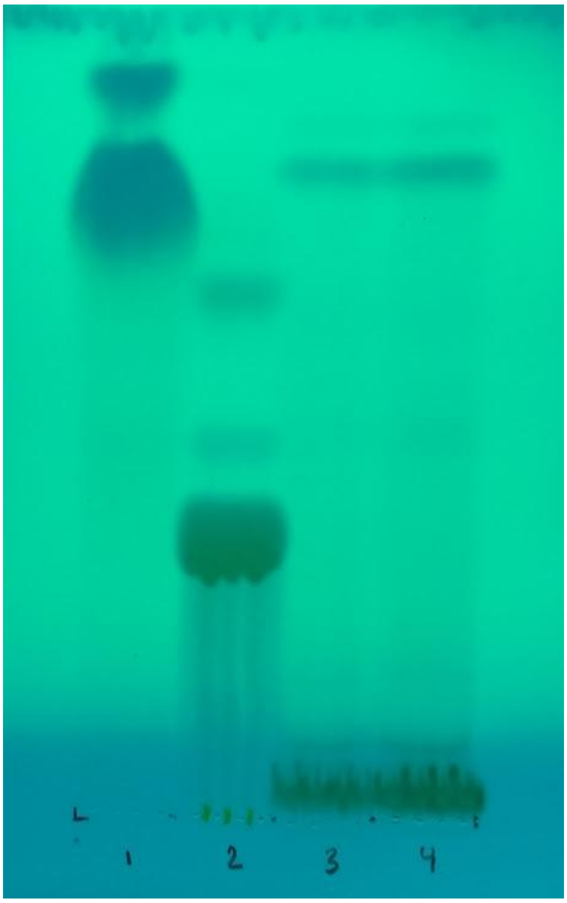

Supplementary figure S1. Thin layer chromatography of leaf aqueous extracts. 1 – Tannic acid (220 µg). 2 – Rutin (220 µg). 3 and 4 – *M. cuspidata* aqueous leaf extract at 4 % (w/v) (70 µg).

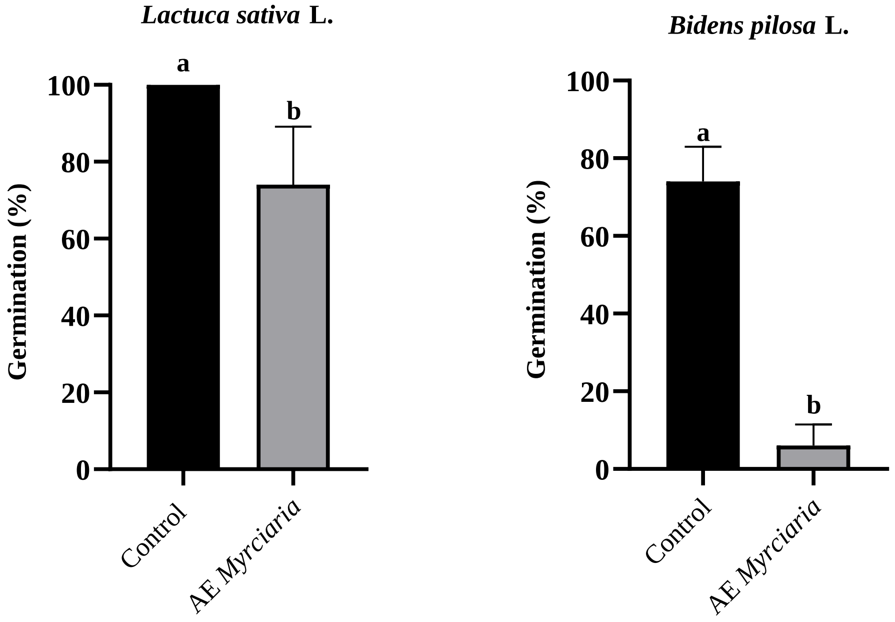

Supplementary figure S2. Final germination percentage *Lactuca sativa* and *Bidens pilosa* on Petri dishes containing *M. cuspidata* leaf aqueous extract 4 % (w/v) or water (control). Treatments were compared using t-test ($p \leq 0.05$).

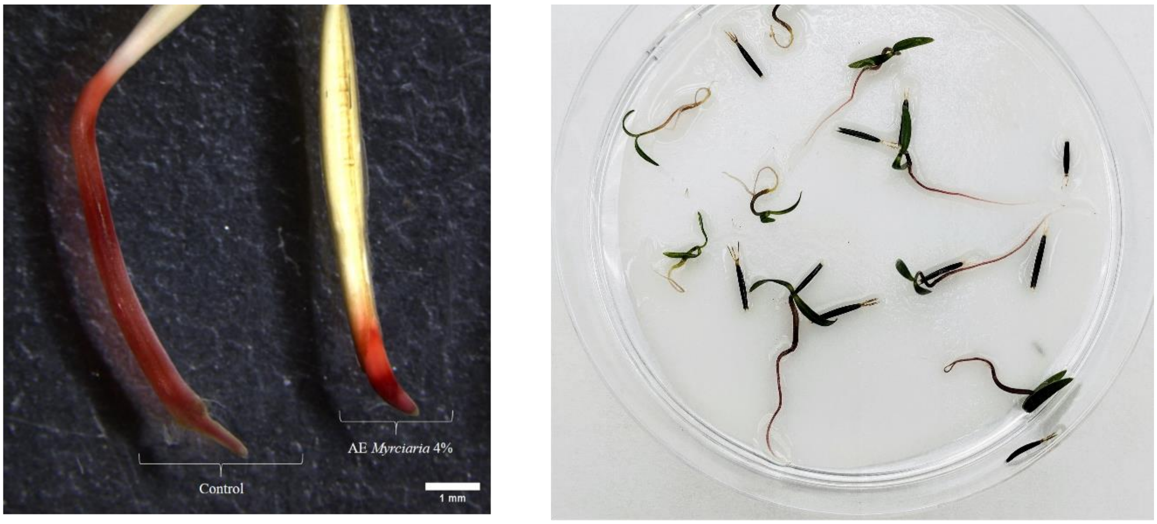

Supplementary figure S3. Excised embryos from achenes of *B. pilosa* after 12 days in Petri dishes with water (control just germinated, right) or *Myrciaria cuspidata* leaf aqueous extract at 4% (w/v) (AE ungerminated, left, same time after sowing of control achenes) subjected to the triphenyltetrazolium chloride test. Alternatively, achenes ungerminated in AE were allowed to germinate after abundant washing in distilled water (right).

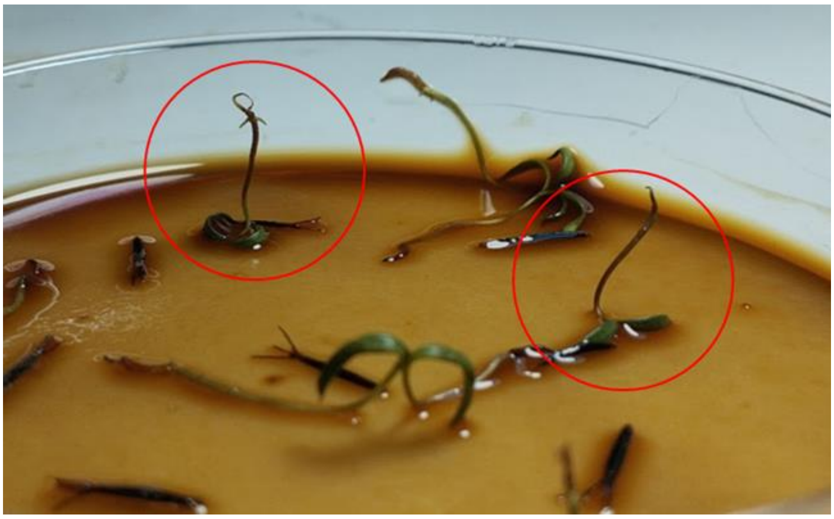

Supplementary figure S4. *Bidens pilosa* seedlings pre-germinated in Petri dishes exposed to *Myrcyaria cuspidata* leaf aqueous extract at 4% (w/v). After radicle protrusion, root of seedlings transferred to plates with 4% (w/v) *Myrcyaria cuspidata* aqueous leaf extract often showed negative gravitropism (red circles).

A B

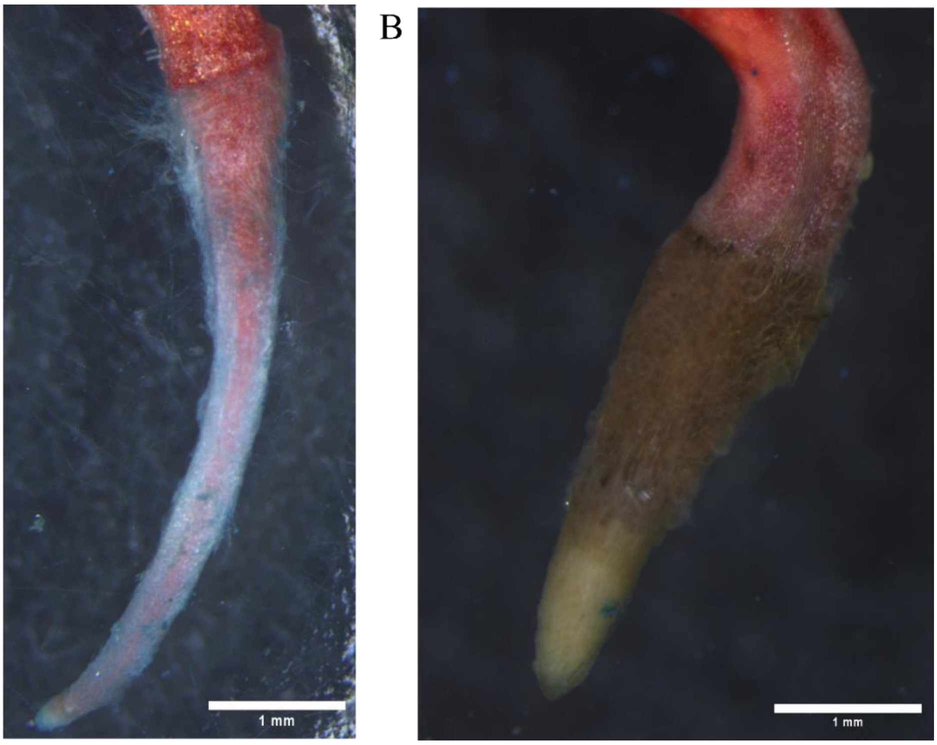

Supplementary figure S5. Root damage in *L. sativa* seedlings. Seeds were pre-germinated and transferred to Petri dishes under control conditions (A) and treated with *Myrciaria cuspidata* leaf aqueous extract at 4% (w/v). (B). A tetrazolium viability test on the roots was performed 6 days after treatment.

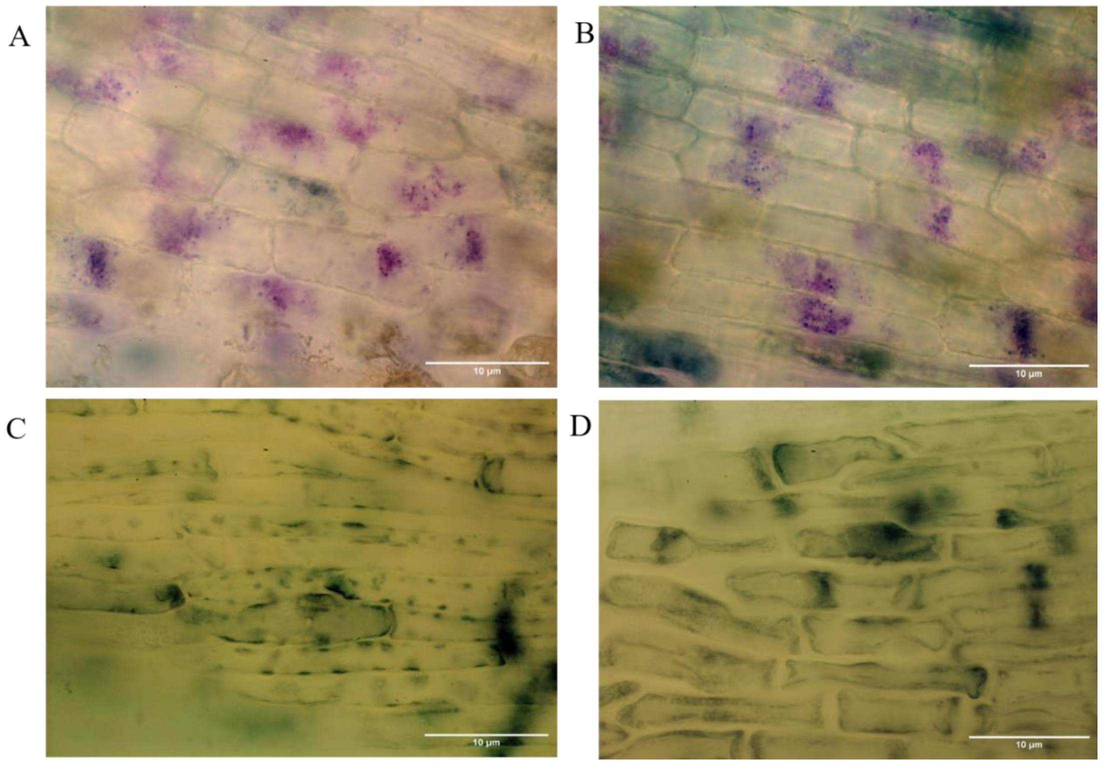

Supplementary figure S6. Mitochondrial damage in *L. sativa* roots. Seeds were pre-germinated and transferred to Petri dishes with water (control) (A, B) or 4% (w/v) leaf aqueous extract of *Myrciaria cuspidata* (C, D). Root mitochondrial viability test with Janus Green was done at day 1 and 2 after transfer to water (A, B) or extract treatment (C, D), respectively. Active mitochondria appear pink, whereas non-functional organelles stain green.

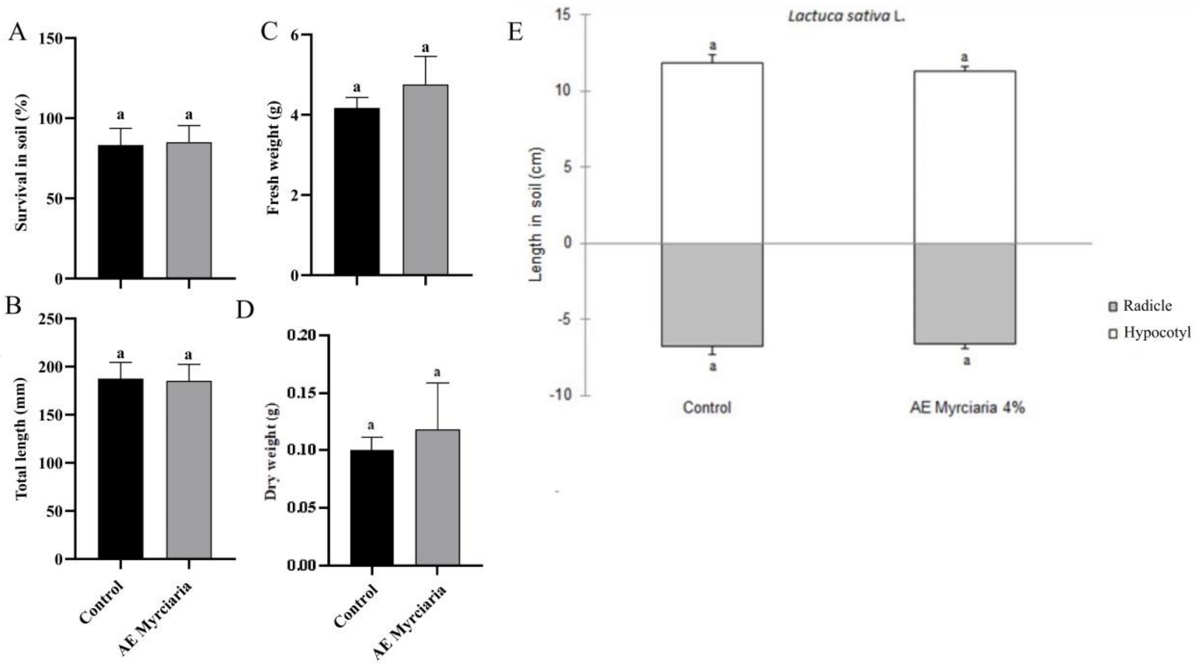

Supplementary figure S7. Growth of pre-germinated *Lactuca sativa* seedlings on solid substrate. Seedlings were sprayed twice (0 and 48h) with water (control) or *Myrciaria cuspidata* leaf aqueous extract at 4% (w/v). Plants were harvested after 15 days from the first application of the treatments. A - Survival of plants in soil; B - Total length; C – fresh weight; D – dry weight; E - length of hypocotyl and radicle. Control and treated groups were compared using t-test ($p \leq 0.05$).

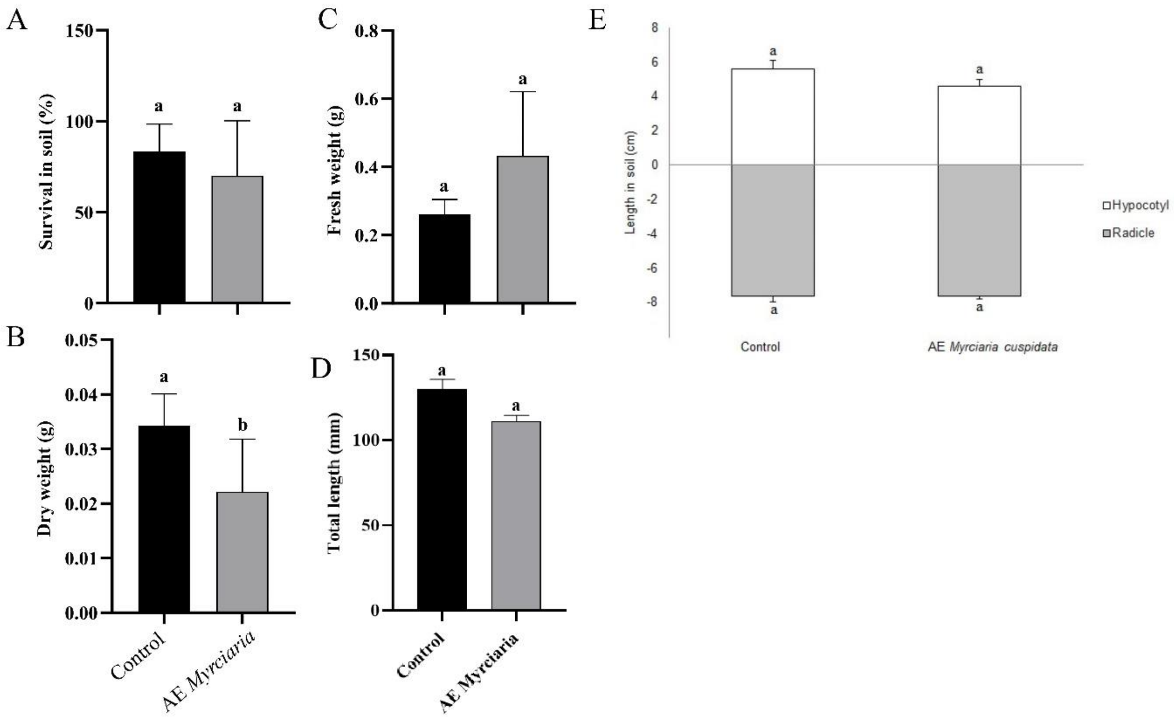

Supplementary figure S8. Growth of pre-germinated *Bidens pilosa* seedlings on solid substrate. Seedlings were sprayed twice (0 and 48h) with water (control) or *Myrciaria cuspidata* leaf aqueous extract at 4% (w/v). Plants were harvested after 15 days from the first application of the treatments. A - Survival of plants in soil; B - Total length; C – fresh weight; D - dry weight; E - length of hypocotyl and radicle. Control and treated groups were compared using t-test ($p \leq 0.05$).
